# The Neural Correlates of Implicit Cognitive Bias Toward Internet-Related Cues in Internet Addiction: An ERP Study

**DOI:** 10.3389/fpsyt.2018.00421

**Published:** 2018-09-07

**Authors:** Limin Chen, Hongliang Zhou, Yue Gu, Shuai Wang, Jun Wang, Lin Tian, Hongmei Zhu, Zhenhe Zhou

**Affiliations:** ^1^Wuxi Mental Health Center, Wuxi, China; ^2^Nanjing Brain Hospital Affiliated to Nanjing Medical University, Wuxi, China; ^3^Basic Medicine College of Nanjing Medical University, Nanjing, China

**Keywords:** internet addiction, implicit cognition, the implicit association test, event-related potentials, internet-related cues

## Abstract

Internet addiction is a sort of non-psychoactive substance dependence. The Implicit Association Test (IAT) is used to measure implicit cognition. Event-related potential (ERP) is one of the most widely used methods in cognitive neuroscience research to investigate the physiological correlates of cognitive activity associated with processing information. Further investigating the ERP characteristics of implicit cognitive bias in Internet addiction would be helpful in understanding the nature of Internet addiction. This study investigated the ERP characteristics of implicit cognitive bias in Internet addiction. The participants included 60 Internet-addicted individuals (IAG) and 60 normal controls (NCG). All participants were measured with ERPs using the IAT. The results showed that there was a significant difference in the Internet-related IAT effect for reaction times between IAG and NCG, and there were stronger positive implicit associations toward Internet related cues in IAG than NCG. Using P1, N2, P3, and N4 as dependent variables, a mixed repeated-measures analysis of variance (ANOVA) on the mean latencies and mean amplitudes revealed a significant interaction between the groups (IAG vs. NCG) and stimulus condition (compatible trials vs. incompatible trials) for the N2 and P3 amplitudes; the simple effects analysis showed that the N2 and P3 amplitudes were larger under the IAG-compatible trial conditions than under the IAG-incompatible trial conditions. In the IAG group, the positive implicit associations with Internet-related cues elicited larger N2 and P3 amplitudes at the occipital lobe sites. These results indicated that Internet addictive individuals show stronger positive implicit associations toward Internet-related cues, and the positive implicit associations toward Internet-related cues elicited ERP changes at occipital lobe sites.

## Introduction

Internet addiction refers to excessive Internet use that has a highly adverse effect on individuals' daily lives. Based on previous studies using neuropsychological and neuroimaging methods, Internet addiction is a sort of non-psychoactive substance dependence (i.e., a type of behavioral addiction) (1–4). To date, there has been an agreement that Internet addiction include four subtypes: Internet gaming, online social networking, Internet pornography, and Internet shopping (5, 6); however, the psychopathological or aetiological mechanism of Internet addiction has been unclear. Using neuropsychological measurements and neuroimaging methods might clarify the nature of Internet addiction.

Implicit cognition is a key term in cognitive psychology; it primarily refers to the perceptual, comprehension, memory, understanding, reasoning, and performance processes that occur through unconscious awareness (7). Previous studies have indicated that some behavior-related associations might be appraised with authenticated associative memory evaluations that get close to and activate pre-existing associations in memory system (8, 9). The Implicit Association Test (IAT) is used to measure implicit cognition. IAT refers to a reaction time-based categorization task that examines the differential associative strength between bipolar targets and appraising attribute concepts as an approach to indexing implicit biases (10). IAT is a commonly used indirect test of association in memory (11, 12). Many studies have reported that implicit cognition is a predictor for some mental disorders, such as alcohol dependence and tobacco dependence (13, 14). For example, previous studies, which have used the IAT to evaluate implicit associations in tobacco, alcohol, marijuana, and cocaine use, have demonstrated that the IAT effectively differentiated substance users from non-users (15–18).

Because of the potential role for psychopathology or etiology, research of implicit cognition has increased, particularly within many mental disorders. A recent study reported that negative associations between Internet addiction and implicit learning abilities (19). To identify the potential mechanisms of dyscontrolled Internet use in individuals with Internet gaming addiction, a study investigated the positive motivational implicit response to Internet gaming cues and concluded that individuals with Internet gaming addiction had a positive motivational implicit response to screenshots of online games; implicit cognition might also be associated with dyscontrolled online gaming (20).

In the past decades, the mechanisms of implicit cognition basis in substance addiction has been evaluated with neuroimaging methods, such as functional magnetic resonance imaging (fMRI) and event-related potentials (ERPs). For example, a previous study assessed activation in the neural substrates involved in implicit associative processes through fMRI of an alcohol-IAT focused on positive outcomes of alcohol use, and the results showed that the striatum is responsible for the mediation of implicit associations underlying habit, and the prefrontal cortex is responsible for the mediation of the controlled behaviors (9). Another study used ERPs to investigate the responses of binge drinkers to alcohol-related pictures and showed that the P100 amplitudes elicited by the alcohol-related pictures were significantly larger than those elicited by the non-alcohol pictures (21).

ERP is one of the most widely used methods in cognitive neuroscience research to investigate the physiological correlates of cognitive activity associated with processing information. In particular, ERP is suited to study item on the speed of neural activity. Further investigating the ERP characteristic of implicit cognitive bias in Internet addiction would be helpful in understanding the nature of Internet addiction. To date, there have been no reported studies examining the ERP characteristics of implicit cognitive bias in Internet addiction. In this study, the participants included an Internet addiction individual group (IAG) and a normal control group (NCG). All participants were measured with ERPs using an Internet information-related IAT. The study investigated the ERP characteristics of implicit cognitive bias in Internet addiction.

## Methods

### Time and setting

This study was conducted at Wuxi Mental Health Center, Jiangsu Province, China, from January 2015 to February 2018.

### Characteristics of the samples

#### Internet addiction group

The diagnostic criteria used for Internet addiction consist of the following five items: (I) individuals with Internet addiction should meet the criteria of the modified Diagnostic Questionnaire for Internet Addiction (22); (II) 18 years of age or older; (III) did not meet the criteria of any of the Diagnostic and Statistical Manual of Mental Disorders-5 (DSM-5) axis I disorders or personality disorders; (IV) not diagnosed with tobacco or alcohol dependence; and (V) not diagnosed with some central nervous systemic diseases. Clinical assessments of all subjects were conducted by two psychiatric residents to collect patient medication and sociodemographic data and to confirm or exclude a DSM-5 diagnostic criterion for any mental illness and a diagnostic criterion for Internet addiction; the duration of each individual's Internet addiction was determined through a retrospective diagnosis. Researchers required the Internet addictive individuals to recall their lifestyles. IAG participants were recruited from the Wuxi Mental Health Center, China. A total of 60 Internet addictive individuals were recruited into the IAG group, including 51 outpatients and 9 inpatients. The reliability of these self-reports from the individuals with Internet addiction was determined by visiting their roommates and intimate friends. Individuals with Internet addiction spent 11.48 h/day (standard deviation = 2.07) on online activities. The duration of being online each week was 6.29 days (standard deviation = 0.57).

#### Normal control group

Normal controls were selected from the local community through local advertisements. All normal controls underwent clinical assessments by two psychiatric residents to collect patient medication and sociodemographic data and to confirm or exclude a DSM-5 diagnostic criterion for any mental illness. Normal controls were tested with the modified Diagnostic Questionnaire for Internet Addiction to exclude a diagnosis of Internet Addiction. Normal controls were excluded from the research if they were substance dependants or were diagnosed with some central nervous systemic diseases. Sixty individuals were matched by sex and age with IAG participants and served as the NCG. Referring to the previous Internet addiction study (3), only normal controls who spent less than 2 h/day on the Internet were placed in the NCG.

Prior to the experiment, a psychiatric associate chief physician re-checked the participants' profiles. All participants' emotional states were tested with the Hamilton Depression Scale (HAMD, 17-item version) and Hamilton Anxiety Scale (HAMA). The Annett handedness scale (3) was used to evaluate all participants' handedness.

The subjects and normal controls received written informed consent forms and provided their own written informed consent to participate in this research. All participants were paid $48.39 plus travel costs. The Ethics Committee of Wuxi Mental Health Center, China, approved the protocol for the research project.

## Neuropsychological test

### Internet-related implicit association test

The subjects and normal controls performed an Internet-related IAT. The Internet-related IAT was referred from an alcohol-IAT that was employed in a previous study by Ames et al. (9). Neither the subjects nor the normal controls received any instructions during the experiment. All participants were asked to go as fast as they could (correctly). The stimuli to be categorized were randomly presented target categories (Internet-related pictures vs. mammal pictures) and attribute categories (positive words vs. neutral words). The target categories (prime stimuli) were six Internet-related pictures and six mammal pictures, and the attribute categories were six positive and six neutral word (two Chinese character words) categories, which were identified through open-ended questionnaires from 180 undergraduate students (40 senior high school students, 101 undergraduate students, and 39 graduate students). Six Internet-related pictures, six mammal pictures, six positive, and six neutral word categories were selected, according to their frequency. Thirty students used a 7-point Likert response format to rate the six Internet-related pictures on their perceived relevance to Internet, and the average score was 6.09 (standard deviation = 0.51). The Internet-related pictures included the WeChat icon, King of Glory (online-game) icon, Taobao icon, Google Chrome icon, Internet explorer icon, and Tencent QQ icon; the mammal pictures included a Dog, Monkey, Horse, Pig, Sheep, and Dolphin. Positive words included Happy, Attractive, Relaxed, Excited, Friendly and Sociable, and neutral words included Common, Calmness, Impartial, Brown, Stationary, and Objective. Thirty students used a 7-point scale ranging from 1 (very approved) to 7 (very disapproved) to rate the affective intensity of six positive and six neutral words; the average score of the Positive words was 6.33 (standard deviation = 0.71), the average score of Neutral words was 3.55 (standard deviation = 0.30).

Combinations of Internet-related picture + positive word vs. mammal + neutral word were compatible trials, while combinations of mammal picture + positive word vs. Internet-related picture + neutral word were incompatible trials.

The target categories (prime stimuli) and the attribute categories were presented on a 17-inch computer monitor using E-Prime 2.0 software. The attribute words (Size 40) and the red “+” (1.0 × 1.0 cm) were presented centrally on the screen.

In this IAT, there were 80 exposures in compatible blocks and 80 in incompatible blocks. Blocks of compatible trials and incompatible trials were counterbalanced, and trials within the blocks were randomly ordered. Fixation point trials were baseline. A red “+” was used in the presentation of the fixation with onset timing ranging from 1.0 to 4.5 s, followed by stimuli presentation. Maximum exposure of test stimuli was for 2 s. There was an intertribal interval (2 s) after a participant pressed a response key, and then the trial was over and followed by the next trial.

**Figure 1:**
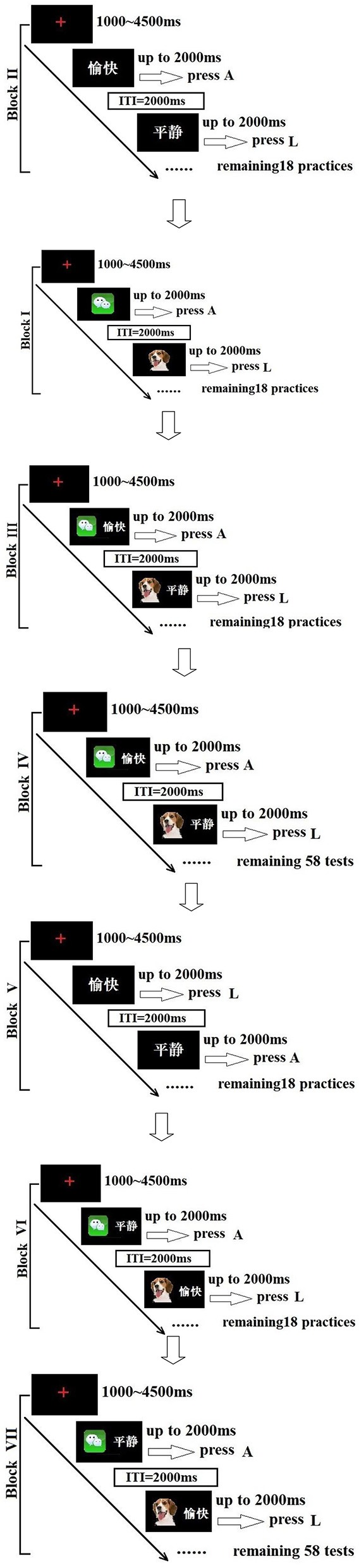
A cartoon illustrating the Internet-related IAT. 愉快, happy; 平静, calmness. ITI, intertribal interval; ms, millisecond.

Referred from Ames et al. (9), the Internet-related IAT consisted of the following blocks: (I) a target category practice (20 trials), during the experiment, all participants were requested to press the A key for the Internet-related picture and press the L key for the mammal picture; (II) an attribute category practice (20 trials), during the experiment, all participants were requested to press the A key for the positive word and press the L key for the neutral word; (III) a compatible block with both target and attribute category practice (20 trials), during the experiment, all participants were requested to press the A key for combinations of the Internet-related picture + the positive word and press the L key for the mammal + neutral word; (IV) a compatible block with both target and attribute category tests (60 trials), during the experiment, all participants were requested to press the A key for combinations of the Internet-related picture + the positive word and press the L key for the mammal + neutral word; (V) a target category only used in the reversed positions practice (20 trials); (VI) an incompatible block with both a reversed target category and the attribute category practice (20 trials); and (VII) an incompatible block with both the reversed target category and the attribute category test (60 trials) (Figure 1). Only the data from block IV and block VII were used for the analysis. According to the previous algorithm used for D-600 measurements (23), the IAG and NCG response latencies were calculated separately.

### Event-related potential measurements

Referencing the international 10/20 system, electroencephalograms were recorded with the Stellate Harmonie Electroencephalogram device (Physiotec Electronics Ltd., Canada) using Electro-Cap Electrode System (ECITM Electro-Caps, Electro-cap International, INL, USA). Combined ear electrodes served as a reference, and the ground electrode was attached to the forehead. Vertical and horizontal electrooculograms were recorded from above and below the right eye and at the right and left outer canthi. The inter-electrode impedance was below 5 kΩ. The band-pass filter was 0.05–100 Hertz (Hz), and the sample rate was 250 Hz. Electroencephalogram and electrooculogram waveforms were filtered with bandpass filter 0.01–40 Hz, 24 dB/oct. The stimulus conditions of the ERPs included following two trials: compatible trials (combinations of the Internet-related picture + positive word vs. mammal + neutral word) and incompatible trials (combinations of the mammal picture + positive word vs. Internet-related picture + Neutral word). The trials in blocks 3, 4, 6, and 7 for Internet-related IAT were used for ERP analysis. The confirmation of ERP components depended on the latency after the stimulus onset, and the ERP components included the peak amplitudes of P1, N2, P3, and N4. ERP data from the following six scalp regions, 14 electrode sites altogether, were analyzed: frontal lobe sites (F3, Fz, and F4); parietal lobe sites (P3, Pz, and P4); central lobe sites (C3, Cz, and C4); left temporal lobe sites (T3) and right temporal lobe sites (T4); and occipital lobe sites (O1, Oz, and O2). The ERP epoch in each stimulus condition was 1000 milliseconds (ms) (including 200 ms before the stimulus onset and 800 ms after the stimulus onset). ERP component P1 was defined as the peak negativity within a 0–150 ms latency window, N2 was defined as the peak negativity within a 150–250 ms latency window, P3 was defined as the peak positivity within a 250–350 ms latency window, and N4 was defined as the peak negativity within a 350–450 ms latency window.

### Statistical analysis

All data were analyzed with Statistical Product and Service Solution 18.0 statistical software (SPSS 18.0, WIN version, Inc., Chicago, IL, USA). Comparisons of the demographic and clinical characteristics (education years, HAMA scores and HAMD scores) between IAG and NCG were performed using independent-sample *t*-tests. Comparisons of handedness between IAG and NCG were performed using chi-squared tests. Comparisons of ERP data between IAG and NCG were performed using mixed repeated measures analysis of variance (ANOVA). The degrees of freedom of the F ratio were corrected, according to the Greenhouse–Geisser method. Least square difference tests were performed as *post-hoc* analyses, if indicated.

## Results

### The demographic and clinical characteristics of the samples

The demographic characteristics of all samples are described in Table 1. There were no significant differences in the sex ratio, mean age, age range, mean education years, and handedness between the two groups. Although the mean scores of HAMA and HAMD of IAG were higher than those of NCG, no significant differences were observed between the two groups.

**Table 1:** Demographic and clinical characteristics of the samples.

	**IAG**	**NCG**	**Test statistic**
Sex ratio (M/F)	60 (32/28)	60 (32/28)	–
Mean age (SD)	23 (5)	23 (5)	–
Handedness (R/M/L)	23/15/22	22/17/21	*x*^2^ = 3.60, *p* = 0.18, NS
Age range	18–28	18–28	–
Education years (SD)	10.3 (2.2)	10.1 (2.2)	*t* = 0.585, *p* = 0.560, NS
Dependence duration (month, SD)	35.1 (11.0)	–	–
HAMA (SD)	9.4 (3.2)	8.4 (2.8)	*t* = 1.762, *p* = 0.081, NS
HAMD (SD)	15.2 (4.8)	13.5 (5.1)	*t* = 1.928, *p* = 0.056, NS

*IAG, Internet addition group; NCG, Normal control group; M, male; F, female; SD, standard deviation; HAMA, Hamilton Anxiety Scale; HAMD, Hamilton Depression Scale; NS, not significant*.

### Internet-related IAT effect

The mean D-600 measure for IAG was 0.3152 (standard deviation = 0.3440), and the mean D-600 measure for NCG was 0.0625 (standard deviation = 0.2063). Accord to an independent sample *t*-test, there was a significant difference in the Internet-related IAT effect for the reaction times between IAG and NCG, and it showed stronger positive implicit associations toward Internet-related cues in IAG than in NCG (*t* = 6.901, *p* = 0.001).

The error rate for IAG was 0.0251 (standard deviation = 0.0187), and the error rates for NCG was 0.0260 (standard deviation = 0.0191). According to an independent sample *t*-test, no significant differences in the error rates for the Internet-related IAT were observed between IAG and NCG (*t* = −0.356, *p* = 0.672).

### Analysis of event-related potential data

**Figure 2:**
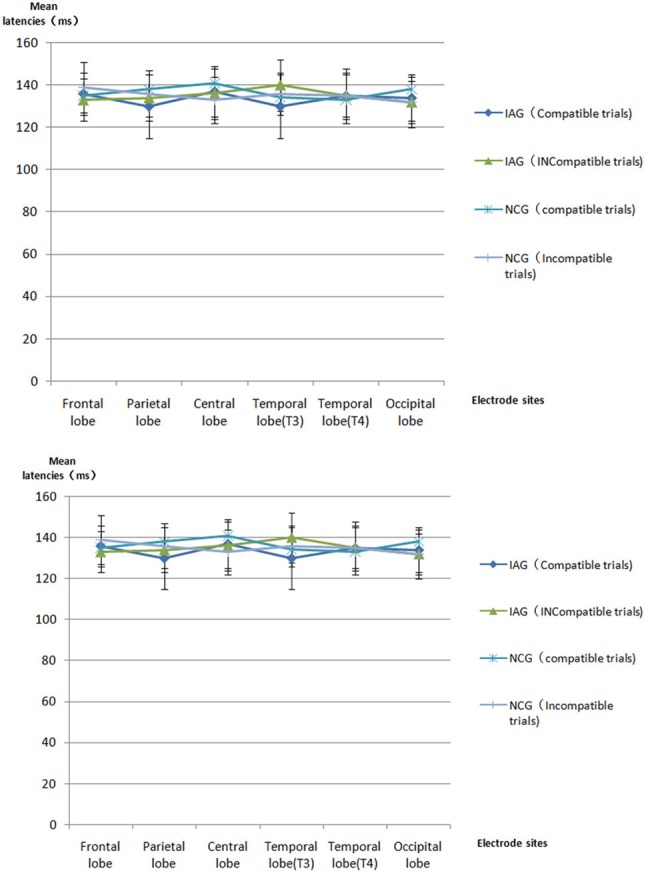
ERP P1 component the latencies and amplitudes.

**Figure 3:**
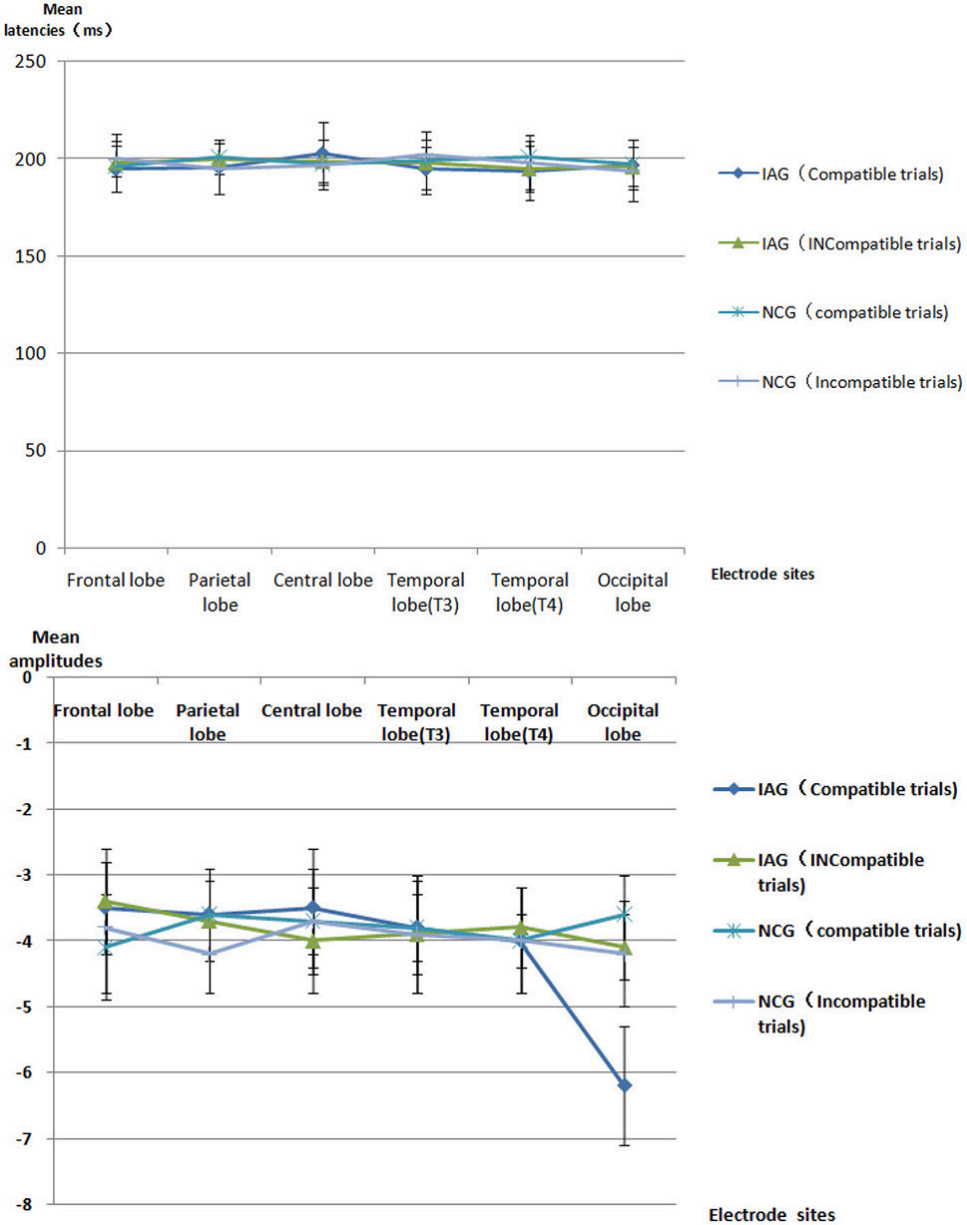
ERP N2 component the latencies and amplitudes.

**Figure 4:**
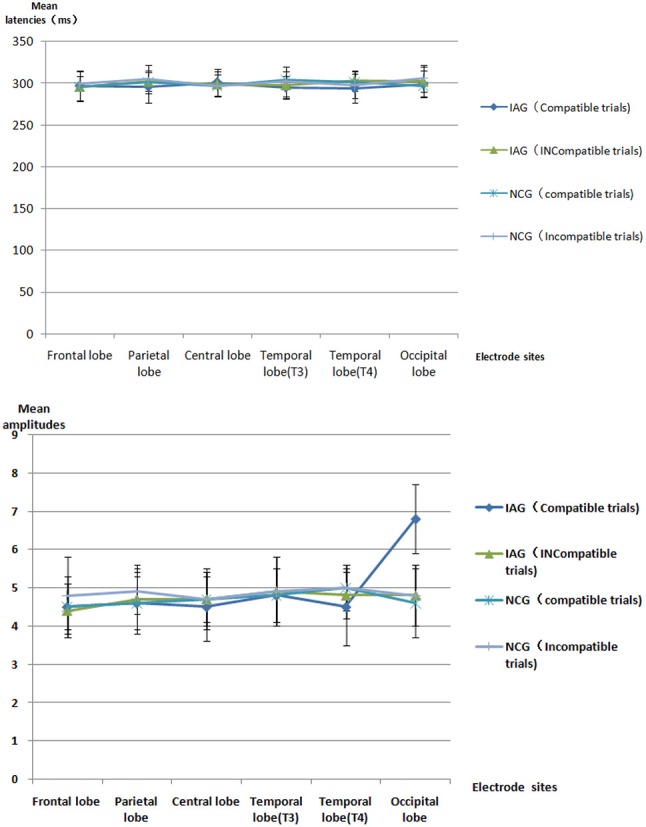
ERP P3 component the latencies and amplitudes.

**Figure 5:**
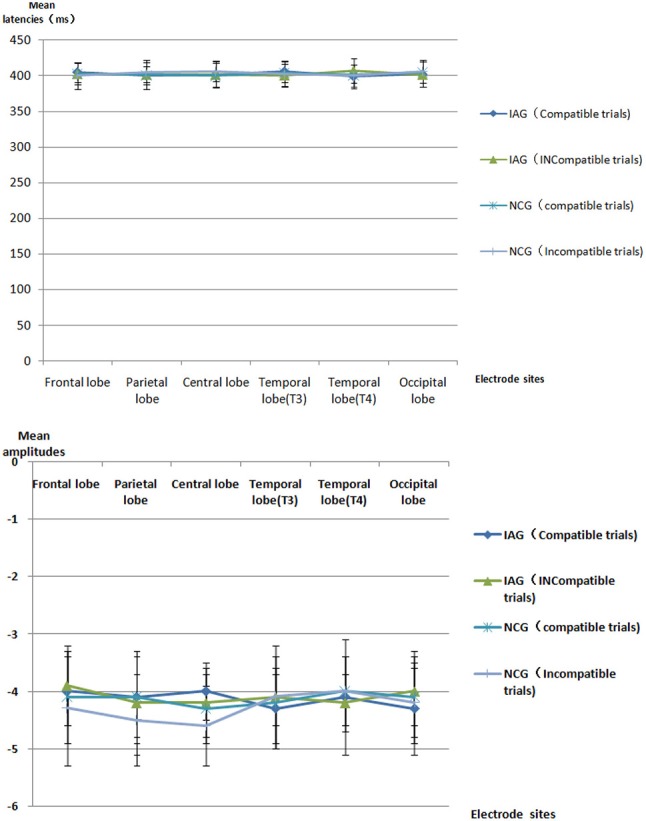
ERP N4 component the latencies and amplitudes.

**Figure 6:**
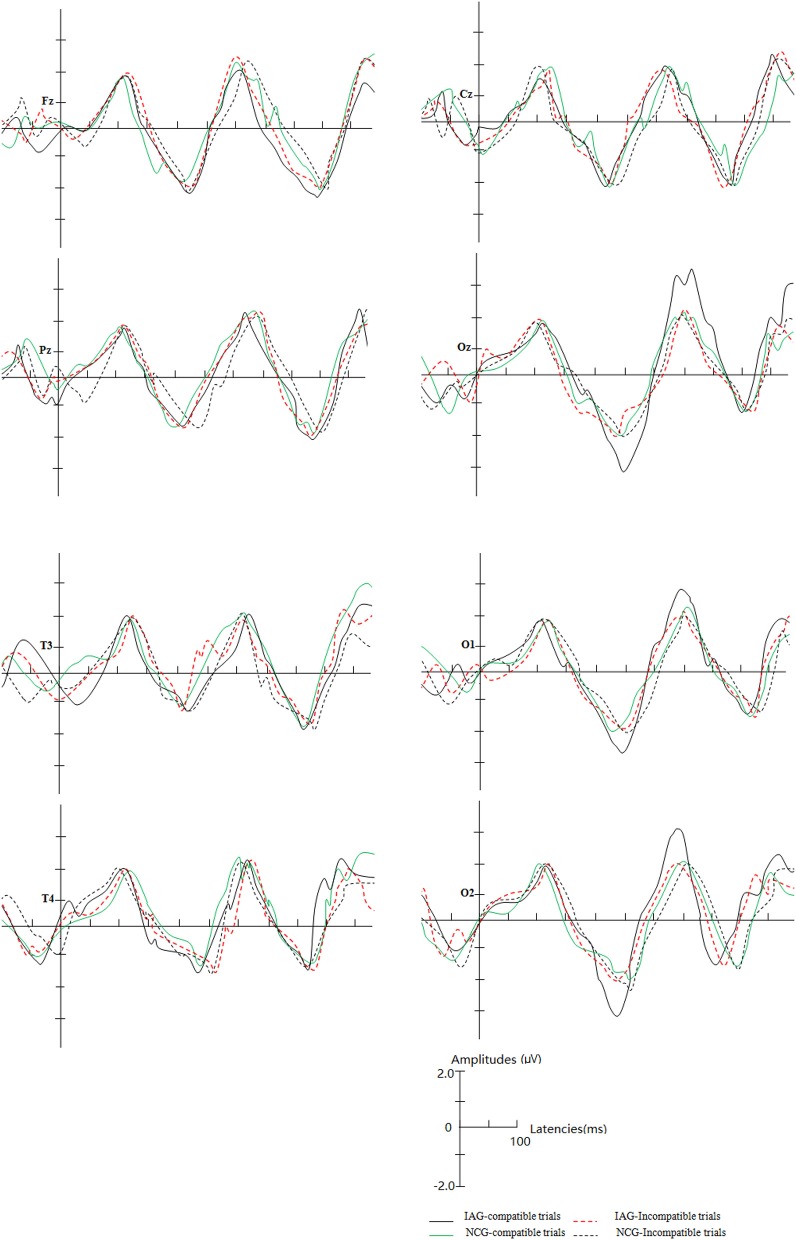
The sketch map of grand average waveforms elicited by IAG-compatible trial stimuli, IAG-incompatible trial stimuli, NCG-compatible trial stimuli, and NCG-incompatible trial stimuli at Fz, Cz, Pz, T3, T4, Oz, O1, and O2. In IAG, at the Oz, O1, and O2 sites, the positive implicit associations with Internet-related cues elicited larger N2 and P3 amplitudes.

The mean latencies and mean amplitudes of ERP component (P1, N2, P3, and N4) of all participants are shown in Tables 2–5 and Figures 2–5. The sketch map of grand average waveforms elicited by IAG-compatible trial stimuli, IAG-incompatible trial stimuli, NCG-compatible trial stimuli, and NCG-incompatible trial stimuli at Fz, Cz, Pz, T3, T4, Oz, O1, and O2 is shown as Figure 6.

**Table 2:** All participants' ERP P1 mean latencies [mean (SD), ms] and mean amplitudes [mean (SD), μV] ^*^.

**Scalp regions**	**IAG**				**NCG**			
	**Compatible trials**		**Incompatible trials**		**Compatible trials**		**Incompatible trials**	
	**Latencies**	**Amplitudes**	**Latencies**	**Amplitudes**	**Latencies**	**Amplitudes**	**Latencies**	**Amplitudes**
Frontal lobe	136 (10)	3.5 (0.4)	133 (10)	3.4 (0.4)	135 (10)	3.3 (0.4)	139 (12)	3.5 (0.3)
Parietal lobe	130 (15)	3.5 (0.5)	134 (9)	3.5 (0.6)	138 (11)	3.5 (0.5)	136 (11)	3.7 (0.6)
Central lobe	137 (12)	3.6 (0.5)	136 (16)	3.3 (0.6)	141 (12)	3.6 (0.4)	133 (11)	3.6 (0.6)
Temporal lobe (T3)	130 (15)	3.4 (0.5)	140 (13)	3.5 (0.5)	134 (12)	3.4 (0.5)	136 (10)	3.3 (0.8)
Temporal lobe (T4)	135 (10)	3.5 (0.4)	135 (10)	3.6 (0.5)	133 (13)	3.5 (0.6)	135 (11)	3.7 (0.6)
Occipital lobe	134 (11)	3.6 (0.7)	132 (11)	3.5 (0.6)	138 (10)	3.3 (0.5)	132 (12)	3.6 (0.6)

* *The sum of all corresponding scalp region latencies and amplitudes divided by the number of electrode sites are the mean latencies and mean amplitudes, respectively*.

**Table 3:** All participants' ERP N2 mean latencies [mean (SD), ms] and mean amplitudes [mean (SD), μV] ^*^.

**Scalp regions**	**IAG**				**NCG**			
	**Compatible trials**		**Incompatible trials**		**Compatible trials**		**Incompatible trials**	
	**Latencies**	**Amplitudes**	**Latencies**	**Amplitudes**	**Latencies**	**Amplitudes**	**Latencies**	**Amplitudes**
Parietal lobe	196 (14)	−3.6 (0.7)	200 (12)	−3.7 (0.6)	201 (8)	−3.6 (0.7)	195 (13)	−4.2 (0.6)
Central lobe	203 (16)	−3.5 (0.9)	199 (10)	−4.0 (0.8)	197 (11)	−3.7 (0.5)	197 (13)	−3.7 (0.8)
Temporal lobe (T3)	195 (11)	−3.8 (0.5)	198 (10)	−3.9 (0.9)	199 (16)	−3.8 (0.7)	202 (8)	−3.9 (0.9)
Temporal lobe (T4)	194 (15)	−4.0 (0.8)	195 (16)	−3.8 (0.6)	201 (12)	−4.0 (0.4)	198 (14)	−4.0 (0.8)
Occipital lobe	197 (13)	−6.2 (0.9)	196 (15)	−4.1 (0.5)	197 (10)	−3.6 (0.6)	194 (16)	−4.2 (0.8)

* *The sum of all corresponding scalp region latencies and amplitudes divided by the number of electrode sites are the mean latencies and mean amplitudes, respectively*.

**Table 4:** All participants' ERP P3 mean latencies [mean (SD), ms] and mean amplitudes [mean (SD), μV] ^*^.

**Scalp regions**	**IAG**				**NCG**			
	**Compatible trials**		**Incompatible trials**		**Compatible trials**		**Incompatible trials**	
	**Latencies**	**Amplitudes**	**Latencies**	**Amplitudes**	**Latencies**	**Amplitudes**	**Latencies**	**Amplitudes**
Frontal lobe	297 (18)	4.5 (0.6)	296 (15)	4.4 (0.7)	296 (18)	4.5 (0.8)	300 (9)	4.8 (1.0)
Parietal lobe	296 (19)	4.6 (0.8)	302 (12)	4.7 (0.9)	301 (11)	4.6 (0.7)	305 (17)	4.9 (0.6)
Central lobe	301 (16)	4.5 (0.9)	299 (17)	4.7 (0.8)	297 (15)	4.7 (0.6)	297 (13)	4.7 (0.7)
Temporal lobe (T3)	295 (14)	4.8 (0.7)	298 (13)	4.9 (0.9)	304 (16)	4.8 (0.7)	302 (18)	4.9 (0.9)
Temporal lobe (T4)	294 (17)	4.5 (1.0)	303 (16)	4.8 (0.6)	301 (12)	5.0 (0.6)	298 (16)	5.0 (0.6)
Occipital lobe	299 (16)	6.8 (0.9)	302 (17)	4.8 (0.8)	297 (18)	4.6 (0.9)	306 (16)	4.8 (0.8)

* *The sum of all corresponding scalp region latencies and amplitudes divided by numbers of electrode sites are the mean latencies and mean amplitudes, respectively*.

**Table 5:** All participants' ERP N4 mean latencies [mean (SD), ms] and mean amplitudes [mean (SD), μV] ^*^.

**Scalp regions**	**IAG**				**NCG**			
	**Compatible trials**		**Incompatible trials**		**Compatible trials**		**Incompatible trials**	
	**Latencies**	**Amplitudes**	**Latencies**	**Amplitudes**	**Latencies**	**Amplitudes**	**Latencies**	**Amplitudes**
Frontal lobe	405 (14)	−4.0 (0.6)	403 (15)	−3.9 (0.7)	403 (15)	−4.1 (0.8)	400 (19)	−4.3 (1.0)
Parietal lobe	400 (19)	−4.1 (0.8)	402 (19)	−4.2 (0.9)	401 (11)	−4.1 (0.7)	405 (17)	−4.5 (0.8)
Central lobe	401 (17)	−4.0 (0.5)	402 (17)	−4.2 (0.6)	400 (19)	−4.3 (0.6)	406 (14)	−4.6 (0.7)
Temporal lobe (T3)	406 (15)	−4.3 (0.6)	401 (13)	−4.1 (0.5)	404 (16)	−4.2 (0.8)	402 (18)	−4.1 (0.9)
Temporal lobe (T4)	399 (17)	−4.1 (1.0)	407 (18)	−4.2 (0.5)	401 (17)	−4.0 (0.6)	400 (16)	−4.0 (0.6)
Occipital lobe	402 (18)	−4.3 (0.8)	402 (17)	−4.0 (0.6)	405 (18)	−4.1 (0.8)	406 (16)	−4.2 (0.6)

* *The sum of all corresponding scalp region latencies and amplitudes divided by the number of electrode sites are the mean latencies and mean amplitudes, respectively*.

Using P1, N2, P3, and N4 as dependent variables, a 2 × 2 × 6 mixed repeated measures ANOVA on the mean latencies and mean amplitudes, with group (IAG vs. NCG) as a between-subject factor and stimulus condition (compatible trials vs. incompatible trials) and scalp regions (frontal lobe, parietal lobe, central lobe, temporal lobe (T3), temporal lobe (T4), and occipital lobe) as within-subjects factors, was performed.

#### P1 component

There were no significant effects for P1 latency and amplitude.

#### N2 component

There were no significant effects for N2 latency. The results revealed a significant interaction between group (IAG vs. NCG) and stimulus condition (compatible trials vs. incompatible trials) [*F*_(1, 119)_ = 32.76, *p* = 0.000]. The simple effects analysis showed that N2 amplitudes were larger under the IAG-compatible trial conditions than under the IAG-incompatible trial conditions [*F*_(1, 119)_ = 5.10, *p* = 0.018]. In IAG, the positive implicit associations toward Internet related cues elicited larger N2 amplitudes. There was a significant three-way interaction between group (IAG vs. NCG), stimulus condition (compatible trials vs. incompatible trials) and scalp regions (frontal lobe, parietal lobe, central lobe, temporal lobe (T3), temporal lobe (T4), and occipital lobe) [*F*_(4, 236)_ = 9.35, *p* = 0.000]. The simple effects analysis showed a significant interaction between group (IAG vs. NCG) and stimulus condition (compatible trials vs. incompatible trials) on the occipital lobe sites [*F*_(1, 119)_ = 29.78, *p* = 0.000]. At the occipital lobe sites, IAG-compatible trials evoked larger N2 amplitudes than IAG-incompatible trials. There were no significant effects in the frontal lobe, parietal lobe, central lobe, temporal lobe (T3), and temporal lobe (T4) sites.

#### P3 component

There were no significant effects for P3 latency. The results revealed a significant interaction between group (IAG vs. NCG) and stimulus condition (compatible trials vs. incompatible trials) [*F*_(1, 119)_ = 35.86, *p* = 0.000]. The simple effects analysis showed that the P3 amplitudes were larger under the IAG-compatible trial conditions than under the IAG-incompatible trial conditions [*F*_(1, 119)_ = 6.47, *p* = 0.025]. In IAG, the positive implicit associations with Internet-related cues elicited larger P3 amplitudes. There was a significant three-way interaction between group (IAG vs. NCG), stimulus condition (compatible trials vs. incompatible trials) and scalp regions (frontal lobe, parietal lobe, central lobe, temporal lobe (T3), temporal lobe (T4), and occipital lobe) [*F*_(4, 236)_ = 8.65, *p* = 0.000]. The simple effects analysis showed a significant interaction between group (IAG vs. NCG) and stimulus condition (compatible trials vs. incompatible trials) at the occipital lobe sites [*F*_(1, 119)_ = 30.42, *p* = 0.000]. At the Occipital lobe sites, IAG-compatible trials evoked larger p3 amplitudes than the IAG-incompatible trials. There were no significant effects in the frontal lobe, parietal lobe, central lobe, temporal lobe (T3), and temporal lobe (T4) sites.

#### N4 component

There were no significant effects for N4 latency and amplitude.

## Discussion

This study is the first to use ERPs to investigate the neural correlates of implicit cognitive bias toward Internet-related cues in Internet addiction. Our study results showed stronger positive implicit associations toward Internet-related cues in IAG than in NCG, and in IAG, the positive implicit associations toward Internet-related cues elicited larger N2 and P3 amplitudes at occipital lobe sites.

Previous studies have indicated that, as a sort of behavioral addiction, Internet addiction shares many psychopathological features with substance dependence (1, 24). Studies of substance dependence have demonstrated that key processes related to reinforcement and cognition in the development and maintenance of substance dependence, particularly the cognition process, represent viable treatment targets for psychosocial and pharmacological interventions (25).

Many scholars have suggested that implicit associations play a crucial role in substance and behavioral addiction (26). In the past decades, many studies, using IAT, have verified whether substance or behavioral addiction present implicit cognition bias. For example, a study used the IAT-Recoding Free (IAT-RF) to measure the predictive validity of recoding-free implicit alcohol associations with positive arousal (27); another previous study, which used IAT modified with pornographic pictures, investigated whether heterosexual male participants have tendencies toward cybersex addiction (26). The above two studies have demonstrated that implicit associations with positive arousal may play a key role in substance and behavioral addiction.

Consistent with a previous study, our results indicated that Internet addictive individuals have tendencies toward Internet related cues.

Event-related potential is a sort of high temporal resolution measures of human brain processing. Because ERPs present the rapid fluctuations associated with the key neurocognitive processes, it is suited to expand our understanding of the underlying neural mechanisms of change during the onset of substance and behavioral addiction (25).

Many studies have investigated the ERP characters when subjects were engaged in an IAT task. In a previous study, two positively valenced stimuli and two negatively valenced stimuli were used as category labels. The results displayed shorter response latencies for compatible trials compared to incompatible trials, and compatible trials tended to generate more positive waveforms in the central and parietal areas compared to incompatible trials (28). A study showed that when the participants performed an IAT task, the recorded ERPs presented an N2 that was larger in the incompatible stimuli, and they deduced that the ERP N2 amplitude reflected greater response monitoring (29). Another study displayed that many brain regions, including medial frontal, cingulate, insular, left-temporal, and parietal cortex, were responsible for ERP N2- and P3-related activity during performed IAT (10).

In this study, under the stimulus conditions of compatible trials, the positive implicit associations toward Internet-related cues elicited larger N2 and P3 amplitudes at occipital lobe sites in Internet addictive individuals. Although the ERP is poor in spatial resolution, it may provide evidence that some cerebral cortices (such as the posterior cingulate cortex) at occipital lobe sites are responsible for the implicit bias toward Internet-related cues in Internet addictive individuals.

Summary, individuals with Internet addiction present stronger positive implicit associations toward Internet-related cues, and the positive implicit associations toward Internet-related cues elicited changes in ERPs (i.e., larger N2 and P3 amplitudes at occipital lobe sites).

Determining the ERP characteristics of implicit cognitive bias in Internet addiction would be helpful in understanding the nature of Internet addiction; furthermore, the results can provide a theoretical basis for the development of possible prevention and treatment strategies for Internet addiction.

This study has some limitations. On the one hand, using the modified Diagnostic Questionnaire for Internet Addiction as a diagnostic tool for Internet addiction is not accurate because its validity as a diagnostic instrument has been not confirmed. On the other hand, to determine the neurotic mechanism of implicit cognitive bias toward Internet-related cues in Internet addiction depends on the integration between temporal resolution and spatial resolution in neuroimaging; however, ERP only provides an excellent temporal resolution. Future studies should use the reliable diagnostic instrument for Internet addiction and fMRI to measure the neurotic mechanism of implicit cognitive bias in Internet addiction.

## Author contributions

ZZ and HZhou designed the study. LC, HZhou, YG, SW, JW, LT, HZhu, and ZZ performed the experiment. LC, HZhou, YG, SW, JW, LT, HZhu, and ZZ analyzed the data and wrote the manuscript. All authors approved the final version of the manuscript for publication.

### Conflict of interest statement

The authors declare that the research was conducted in the absence of any commercial or financial relationships that could be construed as a potential conflict of interest.
